# Custodiol^®^ Supplemented with Synthetic Human Relaxin Decreases Ischemia-Reperfusion Injury after Porcine Kidney Transplantation

**DOI:** 10.3390/ijms222111417

**Published:** 2021-10-22

**Authors:** Augustinas Bausys, Juste Maneikyte, Bettina Leber, Jennifer Weber, Nicole Feldbacher, Kestutis Strupas, Thomas Bernd Dschietzig, Peter Schemmer, Philipp Stiegler

**Affiliations:** 1 General, Visceral and Transplant Surgery, Department of Surgery, Medical University of Graz, 8036 Graz, Austria; abpelikanas@gmail.com (A.B.); juste.maneikyte@gmail.com (J.M.); Jennifer.Weber@medunigraz.at (J.W.); Nicole.Feldbacher@medunigraz.at (N.F.); peter.schemmer@medunigraz.at (P.S.); philipp.stiegler@medunigraz.at (P.S.); 2 Faculty of Medicine, Vilnius University, 01513 Vilnius, Lithuania; kestutis.strupas@santa.lt; 3 Department of Abdominal Surgery, National Cancer Institute, 10224 Vilnius, Lithuania; 4 Relaxera GmbH & Co. KG, 64625 Bensheim, Germany; info@relaxera.com; 5 MHB Medizinische Hochschule Brandenburg, 16816 Neuruppin, Germany

**Keywords:** relaxin, kidney transplantation, apoptosis, ischemia-reperfusion injury, static cold storage

## Abstract

**Objective.** Ischemia-reperfusion injury (IRI) is inevitable after kidney transplantation (KT), impairing outcomes. Relaxin-2 (RLX) is a promising insulin-related peptide hormone that protects against renal IRI in rodents, although large animal models are needed before RLX can be tested in a human setting. **Methods.** In this blinded, randomized, and placebo-controlled experimental study kidneys from 19 donor pigs were retrieved after perfusion with Custodiol^®^ ± RLX (5 or 20 nmol/L) and underwent static cold storage (SCS) for 24 and 48 h, respectively. Subsequently, KT was performed after unilateral right nephrectomy. Study outcomes included markers for kidney function, oxidative stress, lipid peroxidation, and endothelial cell damage. PCR analysis for oxidative stress and apoptosis-related gene panels as well as immunohistochemistry were performed. **Results.** RLX upregulated SOD2 and NFKB expression to 135% (*p* = 0.042) and 125% (*p* = 0.019), respectively, while RIPK1 expression was downregulated to 82% (*p* = 0.016) of corresponding controls. Further RLX significantly downregulated RIPK1 and MLKL expression and decreased the number of Caspase 3- and MPO-positive cells in grafts after SCS. **Conclusions.** RLX supplemented Custodiol^®^ significantly decreased IRI via both antioxidant and anti-apoptotic mechanisms. Clinical trials are warranted to implement synthetic human RLX as a novel additive to preservation solutions against IRI.

## 1. Introduction

Ischemia/reperfusion injury (IRI) is inevitable after kidney transplantation (KT), impairing short- and long-term outcomes [1]. To overcome the shortage of donor kidneys, the use of extended criteria donor (ECD) grafts is increasing, and these organs are known to be more susceptible to IRI [2,3]. Thus, the impact of IRI is an increasing problem, and prevention strategies to better protect grafts are urgently required. Static cold storage (SCS) currently is the simplest, most convenient, and cheapest method of organ preservation in clinical practice [4].

The use of pharmacological supplements for organ preservation solutions targeting specific pathways of IRI is a promising strategy to improve KT outcomes. Relaxin-2 (RLX), an insulin-related peptide hormone, displays antifibrotic, antioxidant, anti-inflammatory, and cytoprotective properties [5,6,7,8], thus it can be considered as a potential substance to reduce IRI. In a rat kidney IRI model, RLX application prior to IR induction resulted in improved renal function, diminished production of pro-inflammatory cytokines, and reduced numbers of apoptotic cells [5,9,10]. Furthermore, RLX demonstrated positive effects against IRI in a series of small animal models of liver, lung, and heart IRI experiments [5]. In the different rodent IRI models cited above, RLX was either given during ischemia and reperfusion or during reperfusion alone. To date, the lack of data from clinically relevant large animal organ transplantation prevents the advancement of RLX into clinical trials. Thus, data presented here are derived from a clinically relevant porcine KT model. This study was performed to evaluate for the first time the impact of RLX supplemented to Custudiol^®^ on a panel of genes involved in oxidative stress and apoptosis, to correlate gene expression with immunohistochemical findings of apoptosis and inflammation, as well as the impact on serum markers of oxidative stress, lipid peroxidation and endothelial cell injury in a porcine KT model.

## 2. Materials & Methods

### 2.1. Study Approval

All animal experiments were performed according to 3Rs, principles of laboratory animal care, and the Austrian national laws. Republic of Austria federal ministry of education, science, and research approval was obtained before this study was started (BMWFW-66.010/0104-WF/V/3b/2016).

### 2.2. Animals 

Nineteen triplets (*n* = 57) of domestic pigs (*sus scrofa domesticus*) weighing from 35 to 50 kg were used for the study. Each triplet consisted of the same generation of siblings-one male pig (donor) and two female pigs (recipients). Experimental animals were kept in the Medical University of Graz animal facility under standard conditions. All the animals had access to water and standard porcine food (PorkoCidKorn F, Garant, Graz, Austria) ad libitum. Before the experiments started, animals were acclimatized for 2 weeks. Blood cross-matching of donor and recipients was tested one week before the experiment started according to standard protocols [11]. Transplantation was performed only if no sign of hemolysis or macro- and microagglutination was observed.

### 2.3. Experimental Design

**Figure 1:**
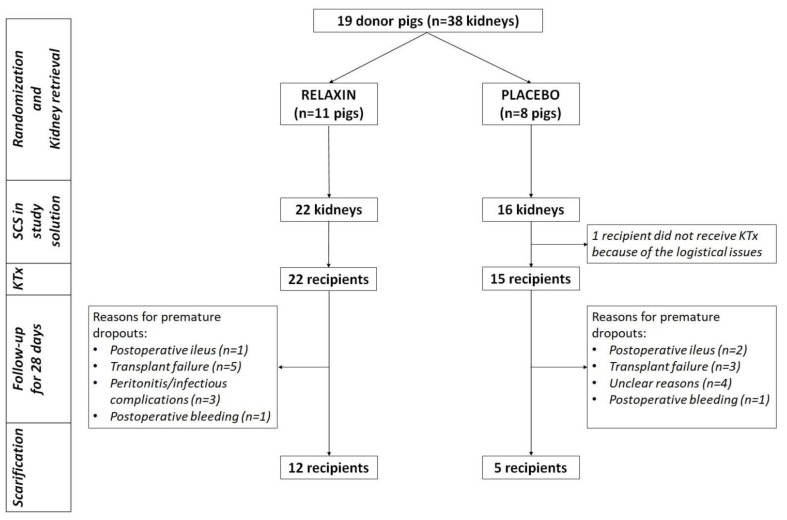
Flowchart study design. SCS: static cold storage; KTx: kidney transplantation.

The study was designed as a blinded, randomized, placebo-controlled trial (Figure 1). Pigs were randomized to RLX and Placebo groups. Custodiol^®^ solution (HTK, Dr. Franz Köhler Chemie GmbH, Bensheim, Germany) supplemented with 5 or 20 nM of Relaxin (Relaxera, Bensheim, Germany) or placebo (5 mg/mL Mannitol with PBS; Relaxera, Bensheim, Germany) were used for organ flushing and preservation, respectively. After randomization, both kidneys were perfused with RLX- or placebo-supplemented preservation solution and retrieved from donor pigs followed by immediate packing of each kidney in 1000 mL of respective study solution and placement on crushed ice for SCS. The right and left kidneys were implanted into randomly assigned sibling recipients after 24 or 48 h of SCS, respectively. No additional RLX was administered for reperfusion. After KT recipient pigs were followed up until the 28th postoperative day (POD) or until premature euthanasia. At the end of the experiment, all recipient pigs were euthanized for organ sampling. The numbers of kidneys in different subgroups of the experiment are presented in supplementary file-Table S1. 

### 2.4. Kidney Perfusion and Retrieval

A midline laparotomy was performed under general anesthesia and the aorta was prepared for cannulation 3 cm below the renal artery. After heparinization (200 IU/kg; i/v) the distal aorta was ligated, and the perfusion catheter was inserted. The proximal aorta was cross-clamped at the level of the aortic hiatus and gravity perfusion with 4000 mL of Custodiol^®^ ± RLX was performed while cooling with crushed ice and cold saline solution before kidney retrieval.

### 2.5. Kidney Implantation

Premedication was performed with midazolam (0.5–1 mg/kg; i/m), ketamine (10–15 mg/kg; i/m) and azaperone (2 mg/kg; i/m). Anesthesia was induced with Propofol (i/v) and maintained by sevoflurane (1–2%) and remifentanil (20–100 µg/kg/h). Antibiotic therapy with Amoxicillin (500 mg; i/v) was administered before skin incision. During anesthesia Ranitidine (50 mg; i/v) and Carprofen (4 mg/kg; i/v) were given for ulcer prevention and pain management. Anticoagulation therapy for donors consisted of heparin (200 IU/kg; i/v) injection before the start of perfusion. Recipients received double therapy with heparin (200 IU/kg; i/v) and aspirin (500 mg; i/v) before revascularization of the donor organ. Under general anesthesia, a midline laparotomy was performed for nephrectomy of the native right kidney. Then, KT was performed starting with the renal vein using an end to side anastomosis with the vena cava inferior using continuous 6/0 Prolene (Ethicon, Somerville, Bridgewater Township, NJ, USA) suture. The arterial anastomosis was performed through an end to side method using continuous 5/0 Prolene (Ethicon, Somerville, Bridgewater Township, NJ, USA) sutures. Following heparinization (200 IU/kg; i/v) organ perfusion was allowed, and renal ischemic time ended. Ureterocystostomy was performed and, after insertion of a double J stent, anastomosis was performed using continuous 3/0 polydioxanone (Ethicon, Somerville, Bridgewater Township, NJ, USA) suture. After precise hemostasis, the abdominal wall was closed.

### 2.6. Immunosuppression and Postoperative Care

Immunosuppression in recipients was induced intraoperatively with two doses of prednisolone (250 mg; i/v) and basiliximab (20 mg; i/v). The second dose of basiliximab (20 mg; i/v) was administered on the 4th postoperative day. Immunosuppression was maintained with daily administered tacrolimus (30 mg; *p*/o) and prednisolone once a week. Tacrolimus concentration in the blood was measured weekly and the dose was changed if necessary. Trough levels were reached within 2 days and ranged from 6–8 ng/dL.

Postoperative care consisted of pain management with fentanyl (100 µg/h; transdermally) for the first week after surgery, later with carprofen (4 mg/kg; s/c) and buprenorphine (5–10 µg/kg) when necessary. Antibiotic therapy with benzylpenicillin/dihydrostreptomycin (100/100 mg; i/m) was given on the first postoperative day, later once a week. Anticoagulation therapy consisted of daily aspirin (100 mg; *p*/o) and weekly depo-heparin (25,000 IU; i/v). Pantoprazole (40 mg; *p*/o) was administered daily for peptic ulcer prevention. Abdominal ultrasound was performed on the 1st POD and later once a week to evaluate the perfusion and survival of the transplanted kidney. Graft failure was defined as graft thrombosis on follow-up ultrasound or if the graft was not viable at the time of explantation.

### 2.7. Tissue and Blood Sampling

Kidney, ureter, renal artery, and vein tissues were sampled after perfusion, after SCS, and at the end of the experiment. Blood sampling was performed immediately before KT, and later on postoperative days (POD): 1, 7, 14, 21, and 28.

### 2.8. Whole Blood and Serum Measurements

Blood biochemistry, electrolytes, and blood gases were measured with the i-STAT system (Abbot, Chicago, IL, USA).

### 2.9. Immunohistochemistry

After sampling, kidney tissue was placed in 4% neutral buffered formalin and transferred to 80% ethanol after 24 h. For immunohistochemistry 3 µm paraffin-embedded sections were prepared according to standard protocols. To evaluate the occurrence of apoptosis anti-caspase 3 antibody (Abcam, Cambridge, UK; dilution 1:200, mouse monoclonal) was used in combination with the UltraVision LP Detection System HRP Polymer (Thermo Fisher Scientific, Waltham, MA, USA) and DAB chromogen (Dako, Via Real Carpinteria, CA, USA). A similar technique was used to evaluate inflammation and oxidative stress by myeloperoxidase (MPO) antibody (Dako, Via Real Carpinteria, CA, USA; dilution 1:800, Rabbit Polyclonal). After staining all slides were scanned and images were viewed using the Aperio ImageScope ver.12.3.2.8013 software (Leica Biosystems Imaging, Wetzlar, Germany). For the semi-quantification of activated Caspase 3 and MPO positive cells, three independent investigators reviewed at least five randomly assigned areas of the slide and quantitatively graded the staining (negative, slightly positive, positive, strongly positive). The results were transferred to a score from 0–3 and the mean of the observations was used for statistical analysis.

### 2.10. qPCR

Kidney tissue samples were snap-frozen and stored in liquid nitrogen until nucleic acid extraction. Tissue (50–100 mg) was homogenized in 1 mL TRIzol reagent in combination with a MagNA Lyser (Roche Diagnostics GmbH, Mannheim, Germany). Isolation of RNA was completed according to the protocol provided by the manufacturer. Quality and quantity of RNA were determined by Nanodrop 2000 (Thermo Fisher Scientific, Waltham, MA, USA). Two micrograms of RNA were used for reverse transcription (High-Capacity cDNA RT Kit; Thermo Fisher Scientific, Waltham, MA, USA) according to the protocol provided by the manufacturer in a final volume of 20 µL.

Real-time PCR amplification and melting analysis were performed based on an already published method [12,13] using a BioRad CFX96 TouchTM System (Bio-Rad Laboratories Ges.m.b.H., Vienna, Austria). Amount of cDNA corresponding to an equivalent of 5 ng RNA was added to a reaction mix containing Promega GoTaq^®^ qPCR Master Mix (Promega, Madison, WI, USA) containing 1 µM of each primer in a final reaction volume of 10 μL. The PCR reaction mixture was subjected to an initial denaturation at 95 °C for 10 s, followed by 45 cycles of denaturation at 95 °C for 10 s, annealing at 58 °C for 20 s, and elongation at 72 °C for 30 s followed by a melting curve (60 to 95 °C). For detailed information on primers used see Table 1.

Gene expression was determined using the Bio-Rad CFX Manager 3.1 (Bio-Rad Laboratories Ges.m.b.H., Vienna, Austria) using the Cq regression method embedded in the program. All PCR reactions were completed in duplicates. Relative gene expression was calculated using multiple reference genes (ACTB and GAPDH) by the Vandesompele method [14,15].

## 3. Statistical Analysis

Statistical analysis was performed using SPSS v.25.0 (SPSS Inc., Chicago, IL, USA). Data are presented as median and quartiles (Q1, Q3) unless stated differently. Differences among groups were analyzed using non-parametric tests-Mann–Whitney U test or Kruskal–Wallis test. For related sample analysis, the Wilcoxon–Signed rank test was used. Graft survival was defined by the Kaplan–Meier method and compared by log-rank test. All statistical tests were 2-sided. *p*-values  <  0.05 were considered statistically significant. To evaluate the effect of 24 or 48 h SCS and treatment with 5 vs. 20 nM of RLX, subgroup analysis was performed.

## 4. Results

### 4.1. Apoptosis/Necroptosis Gene Expression after Kidney Perfusion and Static Cold Storage 

**Figure 2:**
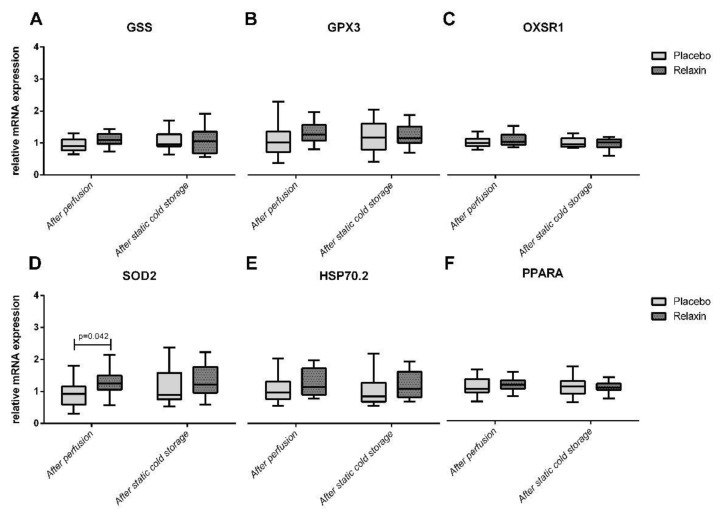
Oxidative stress-related genes expression in kidney tissue after perfusion and static cold storage in Relaxin and Placebo groups. GSS: Glutathione Synthetase; GPX3: Glutathione Peroxidase 3; OXSR1: Oxidative Stress Responsive Kinase 1; SOD2: Superoxide Dismutase 2; HSP70.2: Heat Shock Protein 70.2; PPARA: Peroxisome Proliferators Activated Receptor Alpha.

**Figure 3:**
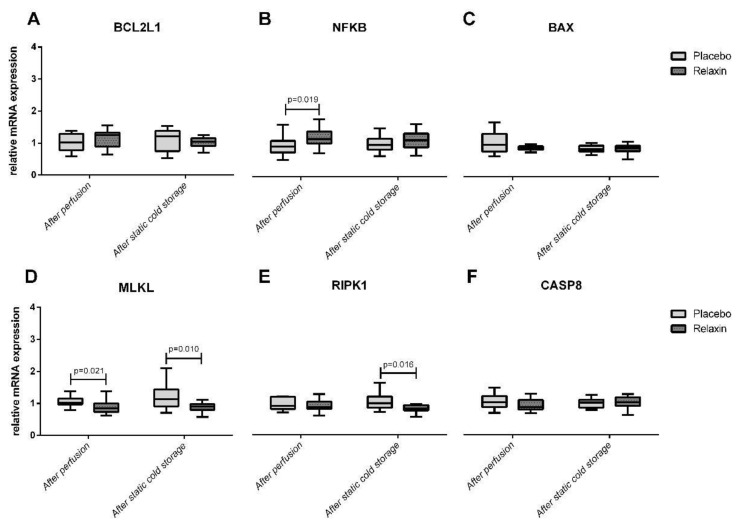
Apoptosis and necroptosis related genes expression in kidney tissue after perfusion and static cold storage in Relaxin and Placebo groups. BCL2L1: BCL2 Like 1; NFKB: Nuclear Factor of Kappa Light Polypeptide Gene Enhancer In B-cells; BAX: BCL2 Associated X Protein; MLKL: Mixed-lineage kinase domain-like protein; RIPKI1: Receptor Interacting Serine/Threonine Kinase 1: CASP8: Caspase 8.

Nineteen donor pigs were randomized to RLX (*n* = 11) or Placebo (*n* = 8) groups. Thirty-eight kidneys (RLX *n* = 22; Placebo = 16) were successfully retrieved after perfusion with Custodiol^®^ ± RLX and placed for SCS in the respective study solution. Perfusion with RLX significantly upregulated SOD2 (Figure 2) and NFKB (Figure 3) expression to 135% (*p* = 0.042) and 125% (*p* = 0.019) of corresponding controls in a placebo group. The MLKL expression after perfusion with RLX was downregulated to 82% of controls (*p* = 0.021) in a placebo group. Furthermore, RLX significantly downregulated RIPK1 and MLKL expression after SCS to 82% (*p* = 0.016) and 81% (*p* = 0.010) of corresponding controls in a placebo group (Figure 3).

### 4.2. Immunohistochemistry for Caspase 3 and MPO in after Kidney Perfusion and Static Cold Storage 

**Figure 4:**
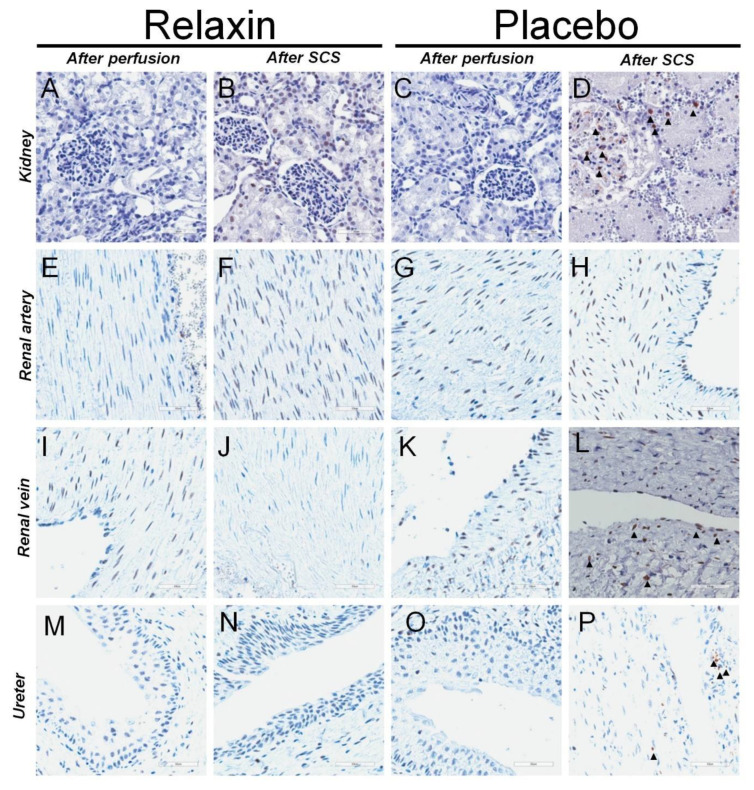
Pictures depicting typical staining for activated Caspase 3 in kidney, renal artery, renal vein, and ureter tissues after perfusion and static cold storage in RLX or control. Representative staining for kidney tissue at 100× magnification for Caspase 3 after perfusion (**A**) and static cold storage (**B**) with RLX and after perfusion (**C**) and static cold storage (**D**) with Custodiol^®^. Caspase 3 positive cells were more common after SCS in a Placebo group. Representative staining for renal artery tissue against Caspase 3 after perfusion (**E**) and static cold storage (**F**) with Custodiol^®^ supplemented with Relaxin or after perfusion (**G**) and static cold storage (**H**) with Placebo. Representative staining for renal vein tissue against Caspase 3 after perfusion (**I**) and static cold storage (**J**) with Relaxin or after perfusion (**K**) and static cold storage (**L**) with Placebo. Caspase 3 positive cells were more common after SCS in a Placebo group. Representative staining for ureter tissue against Caspase 3 after perfusion (**M**) and static cold storage (**N**) with Relaxin or after perfusion (**O**) and static cold storage (**P**) with Placebo. Caspase 3 positive cells were more common after SCS in a Placebo group. All scale bars represent 50 um.

**Figure 5:**
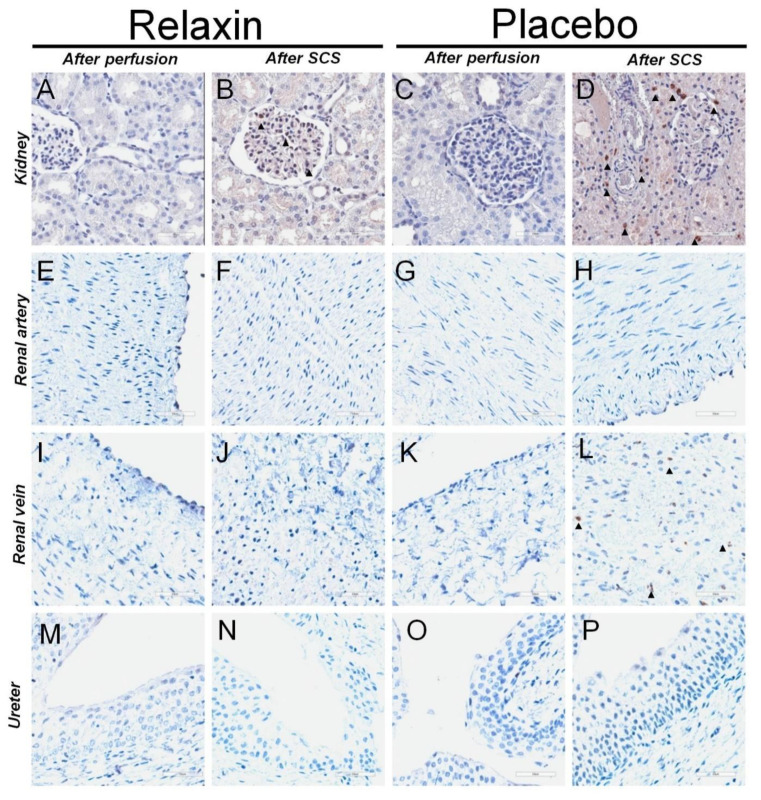
Representative pictures of staining against myeloperoxidase positive cells in kidney, renal artery, renal vein, and ureter tissues after perfusion and static cold storage in Relaxin or Placebo. Representative staining for kidney tissue at 100× magnification against myeloperoxidase positive cells after perfusion (**A**) and static cold storage (**B**) with Relaxin or after perfusion (**C**) and static cold storage (**D**) with Placebo. Myeloperoxidase positive cells were more common after SCS in a Placebo group. Representative staining for renal artery tissue against myeloperoxidase positive cells after perfusion (**E**) and static cold storage (**F**) with Relaxin or after perfusion (**G**) and static cold storage (**H**) with Placebo. Representative staining for renal vein tissue against myeloperoxidase positive cells after perfusion (**I**) and static cold storage (**J**) with Relaxin or after perfusion (**K**) and static cold storage (**L**) with Placebo. Representative staining for ureter tissue against myeloperoxidase positive cells after perfusion (**M**) and static cold storage (**N**) with Relaxin or after perfusion (**O**) and static cold storage (**P**) with Placebo. All scale bars represent 50 um.

The number of Caspase 3 and MPO positive cells after perfusion was similar between placebo and RLX groups (Table 2). However, an increase of apoptotic cells in grafts was observed after SCS. Custodiol^®^ supplemented with RLX prevented the increase of apoptotic cells in kidney, renal vein, and ureter tissue (Table 2). Similarly, Custodiol^®^ supplement with RLX prevented an increase of MPO positive cells in kidneys after SCS. Representative stainings for Caspase 3 and MPO are shown in Figure 4 and Figure 5, respectively.

### 4.3. Kidney Transplantation and Graft Function

**Figure 6:**
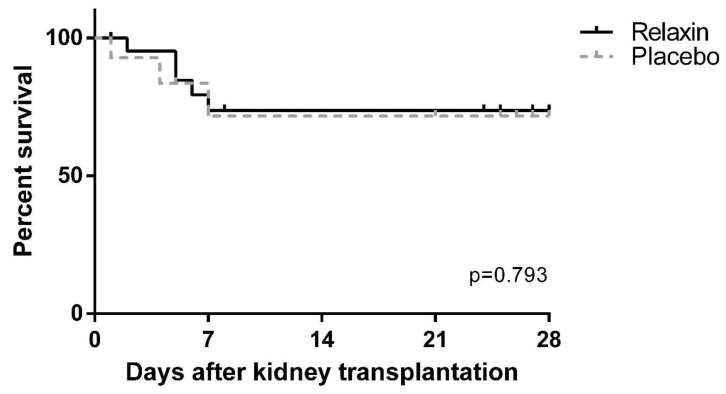
Graft survival after kidney transplantation in Placebo and Relaxin groups.

**Figure 7:**
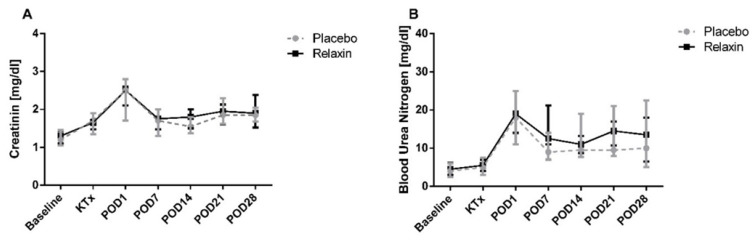
Plasma creatinine (**A**) and blood urea nitrogen (**B**) levels after kidney transplantation in Placebo and Relaxin groups. POD: postoperative day.

After SCS all kidneys were transplanted, except for one organ of the control group, which was not transplanted for logistical reasons. 12 of 22 (54.5%) and 5 of 15 (33.3%) pigs survived until POD 28 in the RLX and placebo groups, respectively (*p* = 0.315). Other animals were sacrificed prematurely because of transplant failure (RLX: *n* = 5; 22.7% vs. Placebo: *n* = 3; 20.0%, *p* = 0.843) or other, transplant non-related postoperative complications (RLX: *n* = 5; 22.7% vs. Placebo: *n* = 7; 46.6%, *p* = 0.126). Graft survival was similar across the study groups (Figure 6). Creatinine and blood urea nitrogen (BUN) peaked on POD1 after KTx, but was similar between RLX and control groups, as remained through the post KTx survival phase (Figure 7).

### 4.4. Subgroup Analysis

#### 4.4.1. 24 vs. 48 h of SCS in Placebo and RLX Groups

Gene expression, immunohistochemistry, graft function representing parameters, and graft survival showed no significant differences among the subgroups of grafts stored for 24 and 48 h within the RLX and placebo groups (Supplementary File).

#### 4.4.2. High vs. Low Concentration of RLX

Gene expression, immunohistochemistry and graft function representing parameters were similar across the animals treated by 5 or 20 nM RLX (Supplementary File).

## 5. Discussion

For the first time, this large animal KT model investigated whether RLX added to conventional Custodiol^®^ affects a panel of genes involved in oxidative stress and apoptosis/necroptosis as well as graft condition in terms of cell death and inflammation after SCS and IRI during organ perfusion. This experimental randomized controlled study demonstrated the beneficial effects of RLX on IRI-related pathways. RLX upregulates the expression of antioxidant (SOD2) and anti-apoptotic (NFkB) genes in kidney grafts after perfusion and downregulates pro-apoptotic/necroptotic (RIPK; MLKL) genes after SCS. Furthermore, RLX added to Custodiol^®^ significantly improved graft condition after SCS by decreasing the number of Caspase 3 and MPO positive cells.

Renal IRI, an inevitable process during KT, is a major trigger for oxidative stress characterized by the overproduction of reactive oxygen species (ROS) leading to cellular damage and graft failure [12,13,14]. Endogenous antioxidant systems are self-defense mechanisms and products of different genes are involved in scavenging ROS or detoxifying enzymes capable of removing ROS-mediated ischemic injury, thus protecting against oxidative stress-induced damage [15,16]. Manganese superoxide dismutase (SOD2), localized in mitochondria, is one of the key enzymes in protecting cells against oxidative stress by catalyzing the dismutation of two superoxide radicals to yield hydrogen peroxide and oxygen [17]. However, the administration of exogenous superoxide dismutase is only partially effective because of poor bioavailability [15,18]. On the other hand, upregulation of SOD protects cells against ROS damage and mediates the apoptosis-suppressive effects [19,20]. As shown in this study, perfusion with Custodiol^®^ supplemented with RLX upregulates SOD2 expression in kidney tissue and thus may improve the graft resistance to oxidative stress-mediated damage.

RLX had no impact on other oxidative stress-related genes investigated herein: GSS is known to detoxify hydrogen peroxide and peroxynitrite by increasing intracellular levels of glutathione [15]. GPX3 is catalyzing the detoxification of hydro- and soluble lipid hydroperoxides by reduced glutathione [21]. OXSR1 is involved in cytoskeleton rearrangements, reaction on osmotic stress, and controlling whether cells proliferate or die by apoptosis [15]. HSP70.2 is a member of the HSP 70 family which is involved in the protection of cells after a potentially lethal stimulus by preventing protein aggregation and facilitating refolding of denatured proteins [15,22]. PPARA which ligands inhibit interleukin-2, TNF-α, and interferon-gamma production by activated T-cells and thus may be involved in acute graft rejection [15,23,24].

Increased SOD2 activity has been shown to prevent cell death via the receptor-mediated apoptotic pathway [25]. Therefore, this study further investigated pro-apoptotic BAX [26] and anti-apoptotic genes including BCL2L1 [27] and NFKB [15] which also influence the inflammatory response in renal injury [28]. As shown in a previous IRI murine model, the pre-activation of NFKB by lipopolysaccharide alleviates the subsequent kidney injury, which is accomplished by the HIF-2α-regulated nitric oxide production [29,30]. In this study, we found upregulated NFKB expression after perfusion with Custodiol^®^ supplemented with RLX. Furthermore, RLX downregulated the expression of RIPK1 and MLKL after SCS. Both of these genes are known to mediate programmed cell death via apoptotic or necroptotic pathways [31,32,33]. RIPK1 and RIPK3 together with necroptotic molecules act as executors and MLKL works as an effector during necroptotic cell death [34]. Recent studies have demonstrated the critical involvement of RIPK and MLKL in IRI [34]. In a transplant setting, RIPK3-mediated necroptosis promotes donor kidney inflammatory injury and reduces allograft survival [35], while blocking necroptosis through RIPK1 benefits in renal IRI [36], therefore the inhibition of RIPK pathway was suggested to be a potentially effective therapy in transplantation [35,37]. Similarly, MLKL is another promising therapeutic target against IRI [34].

To confirm the role of dysregulation of investigated genes we performed immunohistochemical staining for apoptotic and inflammation-induced cell damage to demonstrate that the cascades mentioned above preserved the quality of the kidney graft. We used an antibody against activated Caspase 3 to histologically evaluate apoptotic cell death, because of its reliability and involvement in the final execution phase of apoptosis [15]. Results of the present study demonstrated increased levels of the Caspase 3 positive cells after SCS in the placebo group, while RLX prevented such an increase. The positive effect of Custodiol^®^ supplemented with RLX was documented in most parts of the graft including not only kidney tissue but renal vein and ureter as well. MPO is a neutrophil-derived enzyme able to catalyze the formation of the pro-inflammatory oxidant HOCl and chlorinating species out of H_2_O_2_ and chloride ions [38]. Ischemia stimulates the activation of leukocytes trapped in the kidney vasculature, and such activated neutrophils produce and secrete ROS and hypochlorous acid [39]. These toxic oxygen derivatives exert a strong destructive effect on the cells and tissues [39]. Further, there is in vitro evidence that MPO plays a role in apoptosis by mediating proapoptotic caspase-3 activation [38]. The present study revealed a reduction of increased numbers of MPO-positive cells after SCS by application of RLX as a supplement of Custodiol^®^.

The results of the immunohistochemical analysis were consistent with the results of the gene expression analysis. Together these findings confirmed that RLX as an additive to Custodiol^®^ was beneficial to preserve the kidney graft, as an antioxidant, anti-apoptotic genes were upregulated, pro-apoptotic/necroptotic genes were downregulated and that these changes correlated with the decreased number of Caspase 3 and MPO positive cells in grafts.

RLX did not directly impact renal functional parameters (Creatinine; BUN) after KT. These findings may be related to the design and limitations of the present study. Systemic treatment of recipients with RLX was omitted in the present study and only a minimal amount of peptide was flushed from the graft vascular bed to systemic blood flow. These concentrations are minimal compared to concentrations achieved in studies where 5 µg/kg of RLX was used for effective systemic treatment of recipients at reperfusion [10,40,41]. In addition, healthy and young recipients of our study underwent only unilateral nephrectomy before KT. Thus, the moderate increase in kidney function representing parameters (BUN and Creatinine) after KT may have remained unaffected by the study treatment. The high rate of premature dropouts may be considered a limitation of the present study. However, the long-term follow-up in pig KT models is challenging. Similar to our study, previous experimental studies showed that postoperative mortality may reach up to 25% and up to one-third of animals do not survive 10–60 days after surgery because of various complications [3,42,43].

Nevertheless, despite some limitations, this is the first study that showed the positive effect of RLX against IRI in a large animal KT model. Several previous rodent models showed positive effects of RLX in attenuating IRI in liver, lung, heart, and kidney tissues. RLX reduced the levels of MDA, MPO, and inflammatory cell recruitment; suppressed proinflammatory cytokine programs and increased the expression/activity of the endogenous antioxidant enzymes, such as SOD2, thus preventing cell death by apoptosis [9,10,40,44,45,46,47,48,49]. These results, although very promising, are quite hard to translate to the human setting, highlighting the need for large animal studies. The similarity of swine and humans in size, physiology, urogenital anatomy, immunology, and pathophysiology of diseases make the pig an ideal model for human disease research [50,51]. Nowadays, large animal models are crucial to test medical products such as preservation solutions for transplantation [50], before the novel substances can be investigated in clinical studies. Therefore, the present study is important for the further development of RLX as a substance ameliorating IRI in the future.

## 6. Conclusions

In conclusion, this study revealed for the first time in a large animal model of clinically relevant KT that RLX added to Custodiol^®^ upregulates antioxidant and anti-apoptotic genes and downregulates pro-apoptotic/necroptotic genes. The protective effect of RLX supplemented Custodiol^®^ most likely includes apoptotic pathways and oxidative stress correlating with decreased numbers of Caspase 3 and MPO positive cells in kidney grafts, respectively. Clinical trials are warranted to implement RLX as a novel additive to preservation solutions diminishing IRI in human transplantation.

## Figures and Tables

**Table 1:** Genes used for quantification and primer information.

Accession Number	Forward (5′- > 3′)	Reverse (5′- > 3′)	Product Length
**ACTB (Actin Beta)**			
XM_021086047.1 XM_003124280.5	CTCCAGAGCGCAAGTACTCC	ACTCCTGCTTGCTGATCCAC	90 bp
**GAPDH (Glyceraldehyde-3-Phosphate Dehydrogenase)**			
NM_001206359.1	CCGTGTGTTCCGTGCATTG	GCCAAATCCGTTCACTCCGA	71 bp
**GSS (Glutathione Synthetase)** [15]			
NM_001244625.1	AAGAAGCTGCCAAGATCCTC	ATTCTCTATGGCACGCTGGT	155 bp
**GPX3 (Glutathione Peroxidase 3)** [15]			
NM_001115155.1	GAGACAACTCGGAGATTCTG	GGAACGTGTAGAACTTCTGC	126 bp
**OXSR1 (Oxidative Stress Responsive Kinase 1)** [15]			
NM_214342.1	CCGAAGTTATGGAACAGGTC	GATCATTCTGCAGTGTCAGC	147 bp
**SOD2 (Superoxide Dismutase 2)** [15]			
NM_214127	CCTACGTGAACAACCTGAAC	GATACAGCGGTCAACTTCTC	247 bp
**HSP70.2 (Heat Shock Protein 70.2)** [15]			
NM_213766.1	AGGTGCAGGTGAGCTACAAG	CTGCGAGTCGTTGAAGTAGG	158 bp
**PPARA (Peroxisome Proliferators Activated Receptor Alpha)** [15]			
NM_001044526.1	TGAAGTTCAATGCGCTGGAG	TTGAGCACATGCACGATACC	139 bp
**BCL2 L1 (BCL2 Like 1)** [15]			
NM_214285.1	TGAGTCGGATCGCAACTTGG	ATCGGTTGAAGCGTTCCTGG	150 bp
**NFKB1 (Nuclear Factor of Kappa Light Polypeptide Gene Enhancer in B-cells)** [15]			
NM_001048232.1	GAGGTGCATCTGACGTATTC	CACATCTCCTGTCACTGCAT	138 bp
**BAX (BCL2 Associated X Protein)** [15]			
XM_003127290	GCTGACGGCAACTTCAACTG	CCGATCTCGAAGGAAGTCCA	141 bp
**MLKL (Mixed-lineage kinase domain-like protein)**			
XM_003481791.4	TTGGAAAACACCACGAGGGA	CCCTTCTTGGGTTTGTGTGC	77 bp
**RIPK1 (Receptor Interacting Serine/Threonine Kinase 1)**			
XM_005665536	CACTCGGAGAAATCAAGGCAG	CTGCGCCCTGATGGTTACAAAA	86 bp
**CASP8 (Caspase 8)**			
NM_001031779.2	CCAGGATTTGCCTCCGGTTA	TCACTGTCCAAATGTTCCCCA	99 bp

**Table 2:** Immunohistochemical evaluation for activated Caspase-3 positivity as well as Myeloperoxidase positivity in kidneys after perfusion and static cold storage in Placebo and Relaxin groups. Values are median (Quartile 1; Quartile 3).

		Caspase 3			Myeloperoxidase		
		RLX	Placebo	*p* Value	RLX	Placebo	*p* Value
**Kidney**	After perfusion	0 (0; 0)	0 (0; 0)	0.999	0 (0; 0)	0 (0; 0)	0.999
	After SCS	0 (0; 0.5)	1 (0; 1.6)	0.005	0 (0; 0.5)	1 (0; 1.3)	0.017
**Renal artery**	After perfusion	0 (0; 0)	0 (0; 0)	0.999	0 (0; 0)	0 (0; 0)	0.999
	After SCS	0 (0; 0)	0 (0; 0.2)	0.650	0 (0; 0)	0 (0; 0)	0.660
**Renal vein**	After perfusion	0 (0; 0)	0 (0; 0)	0.811	0 (0; 0)	0 (0; 0)	0.999
	After SCS	0 (0; 0)	0.8 (0; 1)	0.027	0 (0; 0)	0 (0; 1)	0.066
**Ureter**	After perfusion	0 (0; 0)	0 (0; 0)	0.999	0 (0; 0)	0 (0; 0)	0.999
	After SCS	0 (0; 0)	0.6 (0; 1)	0.019	0 (0; 0)	0 (0; 0)	0.839

## Data Availability

The data that support the findings of this study are available from the corresponding author, [B.L.], upon reasonable request.

## Supplementary Materials

The following are available online at https://www.mdpi.com/article/10.3390/ijms222111417/s1, Table S1: The numbers of kidneys in different subgroups. SCS: static cold storage, Table S2. Immunohistochemical evaluation for activated Caspase-3 positivity as well as Myeloperoxidase positivity in kidneys after perfusion and static cold storage for 24 and 48 h in Placebo and Relaxin groups. Values are median (Quartile 1; Quartile 3); Figure S1: Oxidative stress-related genes expression in kidney tissue after perfusion and static cold storage in Relaxin and Placebo groups for 24 and 48 h. GSS: Glutathione Synthetase; GPX3: Glutathione Peroxidase 3; OXSR1: Oxidative Stress Responsive Kinase 1; SOD2: Superoxide Dismutase 2; HSP70.2: Heat Shock Protein 70.2; PPARA: Peroxisome Proliferators Activated Receptor Alpha, Figure S2: Apoptosis and necroptosis related genes expression in kidney tissue after perfusion and static cold storage in Relaxin and Placebo groups for 24 and 48 h. BCL2L1: BCL2 Like 1; NFKB: Nuclear Factor of Kappa Light Polypeptide Gene Enhancer In B-cells; BAX: BCL2 Associated X Protein; MLKL: Mixed-lineage kinase domain-like protein; RIPKI1: Receptor Interacting Serine/Threonine Kinase 1: CASP8: Caspase 8, Figure S3: Plasma creatinine (A) and blood urea nitrogen (B) levels after kidney transplantation in Placebo and Relaxin subgroups stored for 24 and 48 h. POD: postoperative day, Figure S4:. Graft survival after kidney transplantation in Placebo and Relaxin subgroups stored for 24 and 48 h, Figure S5: Oxidative stress-related genes expression in kidney tissue after perfusion and static cold storage in Placebo, Relaxin 5 nM and Relaxin 20 nM subgroups. GSS: Glutathione Synthetase; GPX3: Glutathione Peroxidase 3; OXSR1: Oxidative Stress Responsive Kinase 1; SOD2: Superoxide Dismutase 2; HSP70.2: Heat Shock Protein 70.2; PPARA: Peroxisome Proliferators Activated Receptor Alpha, Figure S6: Apoptosis and necroptosis related genes expression in kidney tissue after perfusion and static cold storage in Placebo, Relaxin 5 nM and Relaxin 20 nM subgroups. BCL2L1: BCL2 Like 1; NFKB: Nuclear Factor of Kappa Light Polypeptide Gene Enhancer In B-cells; BAX: BCL2 Associated X Protein; MLKL: Mixed-lineage kinase domain-like protein; RIPKI1: Receptor Interacting Serine/Threonine Kinase 1: CASP8: Caspase 8. Table S3. Immunohistochemical evaluation for activated Caspase-3 positivity as well as Myeloperoxidase positivity in kidneys after perfusion and static cold storage in Placebo, Relaxin 5 nM and Relaxin 20 nM subgroups. Values are median (Quartile 1; Quartile 3); Figure S7: Plasma creatinine (A) and blood urea nitrogen (B) levels after kidney transplantation in Placebo, Relaxin 5 nM and Relaxin 20nM subgroups. POD: postoperative day, Figure S8: Graft survival after kidney transplantation in Placebo, Relaxin 5 nM and Relaxin 20 nM subgroups.

## Abbreviations

IRI	Ischemia/reperfusion injury
ECD	extended criteria donor
SCS	Static cold storage
RLX	Relaxin-2
POD	postoperative day
PCR	polymerase chain reaction
BUN	blood urea nitrogen
MPO	myeloperoxidase
ACTB	Actin Beta
GAPDH	Glyceraldehyde-3-Phosphate Dehydrogenase
GSS	Glutathione Synthetase
GPX3	Glutathione Peroxidase 3
OXSR1	Oxidative Stress Responsive Kinase 1
SOD2	Superoxide Dismutase
HSP70.2	Heat Shock Protein 70.2
BCL2L1	BCL2 Like 1
PPARA	Peroxisome Proliferators Activated Receptor Alpha
NFKB1	Nuclear Factor of Kappa Light Polypeptide Gene Enhancer in B-cells
MLKL	Mixed-lineage kinase domain-like protein)
BAX	BCL2 Associated X Protein
RIPK1	Receptor Interacting Serine/Threonine Kinase 1
CASP8	Caspase 8
