# Supplementary material for: Custodiol® Supplemented with Synthetic Human Relaxin Decreases Ischemia-Reperfusion Injury after Porcine Kidney Transplantation
Source: Int J Mol Sci. 2021 Oct 22;22(21):11417. doi: 10.3390/ijms222111417 (PMC8583819; doi:10.3390/ijms222111417)
Supplement: Supplementary file 1 [file ijms-22-11417-s001.zip › ijms-1409033-supplementary.pdf]

Supplemetary File

1. ***Number of animals in different subgroups of the experiment***

Placebo, 5nM Relaxin and 20 nM Relaxin were used for organ flushing and preservation in 8, 5 and 6 donor pigs, respectively. The numbers of kidneys in different subgroups of the experiment are presented in Table S1.

**Subgroups of the experiment**	**Number of animals, n**
Placebo 24 h SCS	n=8
Placebo 48 h SCS	n=8
Relaxin 5nM 24 h SCS	n=5
Relaxin 5nM 48 h SCS	n=5
Relaxin 20nM 24 h SCS	n=6
Relaxin 20nM 48 h SCS	n=6

Table S1. The numbers of kidneys in different subgroups. SCS: static cold storage.

2. ***Subgroup analysis***

2.1 24. vs. 48 hours of SCS in Placebo and RLX groups

Oxidative stress-related genes (Figure S1) and apotosis, necroptosis-related genes (Figure S2) expression, immunohistochemistry (Table S1), graft function representing parameters (Figure S3) and graft survival (Figure S4) was similar between the subgroups of grafts stored for 24 and 48 hours within the RLX and placebo groups.

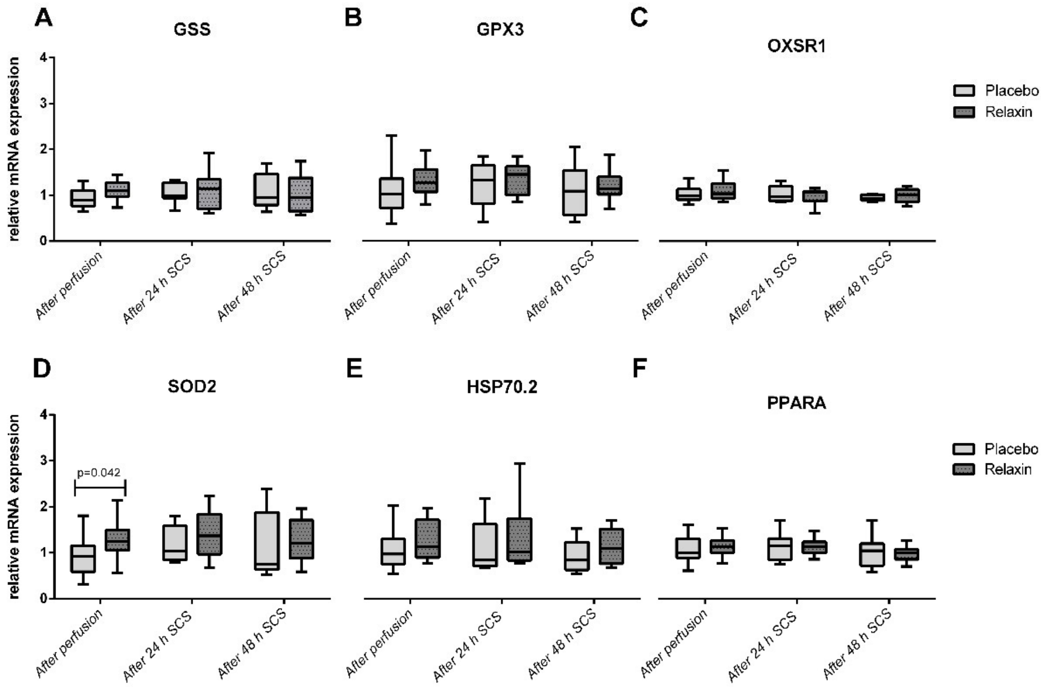

Figure S1. Oxidative stress-related genes expression in kidney tissue after perfusion and static cold storage in Relaxin and Placebo groups for 24 and 48 hours. GSS: Glutathione Synthetase; GPX3: Glutathione Peroxidase 3; OXSR1: Oxidative Stress Responsive Kinase 1; SOD2: Superoxide Dismutase 2; HSP70.2: Heat Shock Protein 70.2; PPARA: Peroxisome Proliferators Activated Receptor Alpha.

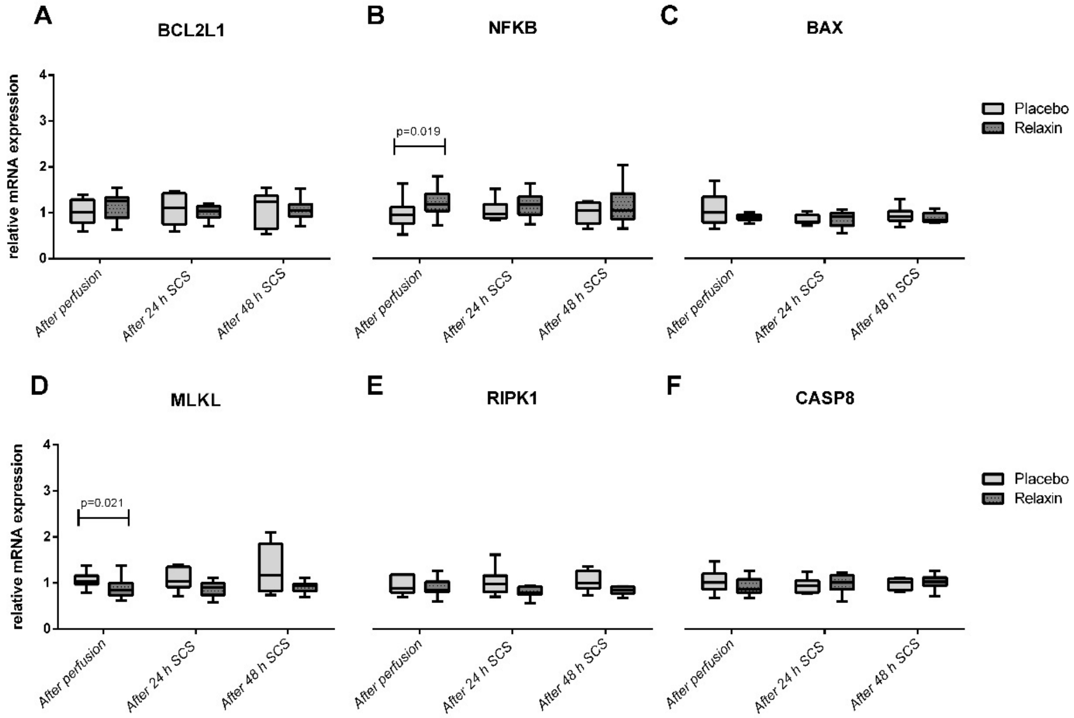

Figure S2. Apoptosis and necroptosis related genes expression in kidney tissue after perfusion and static cold storage in Relaxin and Placebo groups for 24 and 48 hours. BCL2L1: BCL2 Like 1; NFKB: Nuclear Factor of Kappa Light Polypeptide Gene Enhancer In B-cells; BAX: BCL2 Associated X Protein; MLKL: Mixed-lineage kinase domain-like protein; RIPKI1: Receptor Interacting Serine/Threonine Kinase 1: CASP8: Caspase 8.

		**Caspase 3**			**Myeloperoxidase**		
		RLX	Placebo	p value	RLX	Placebo	p value
Kidney	After perfusion	0 (0; 0)	0 (0; 0)	*0.999*	0 (0; 0)	0 (0; 0)	*0.999*
	After 24 hours SCS	0 (0; 1)	1 (0.2; 1.5)	*0.068*	0 (0; 0)	0.5 (0; 1.25)	*0.177*
	After 48 hours SCS	0 (0; 0)	1.1 (0; 1.9)	*0.051*	0 (0; 1)	1 (0.25; 1.5)	*0.062*
Renal artery	After perfusion	0 (0; 0)	0 (0; 0)	*0.999*	0 (0; 0)	0 (0; 0)	*0.999*
	After 24 hours SCS	0 (0; 0)	0 (0; 0.5)	*0.768*	0 (0; 0)	0 (0; 0)	*0.999*
	After 48 hours SCS	0 (0; 0)	0 (0; 0.6)	*0.768*	0 (0; 0)	0 (0; 0.5)	*0.594*
Renal vein	After perfusion	0 (0; 0)	0 (0; 0)	*0.811*	0 (0; 0)	0 (0; 0)	*0.999*
	After 24 hours SCS	0 (0; 0.1)	0 (0; 1)	*0.513*	0 (0; 0)	0 (0; 1.1)	*0.284*
	After 48 hours SCS	0 (0; 0)	1 (0.3; 1)	*0.019*	0 (0; 0)	0.5 (0; 1)	*0.199*
Ureter	After perfusion	0 (0; 0)	0 (0; 0)	*0.999*	0 (0; 0)	0 (0; 0)	*0.999*
	After 24 hours SCS	0 (0; 0)	0 (0; 1)	*0.240*	0 (0; 0)	0 (0; 0)	*0.768*

	After 48 hours SCS	0 (0; 0)	0.8 (0.1; 2)	*0.036*	0 (0; 0)	0 (0; 0.5)	*0.606*

Table S2. Immunohistochemical evaluation for activated Caspase-3 positivity as well as Myeloperoxidase positivity in kidneys after perfusion and static cold storage for 24 and 48 hours in Placebo and Relaxin groups. Values are median (Quartile 1; Quartile 3);

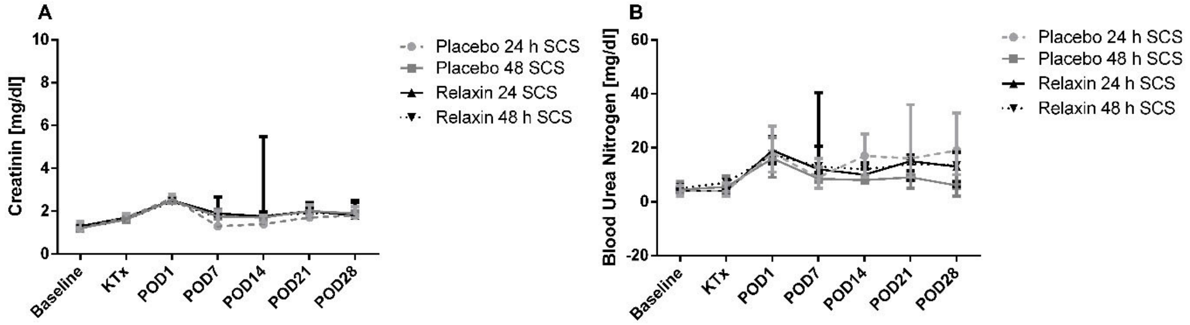

Figure S3. Plasma creatinine (A) and blood urea nitrogen (B) levels after kidney transplantation in Placebo and Relaxin subgroups stored for 24 and 48 hours. POD: postoperative day.

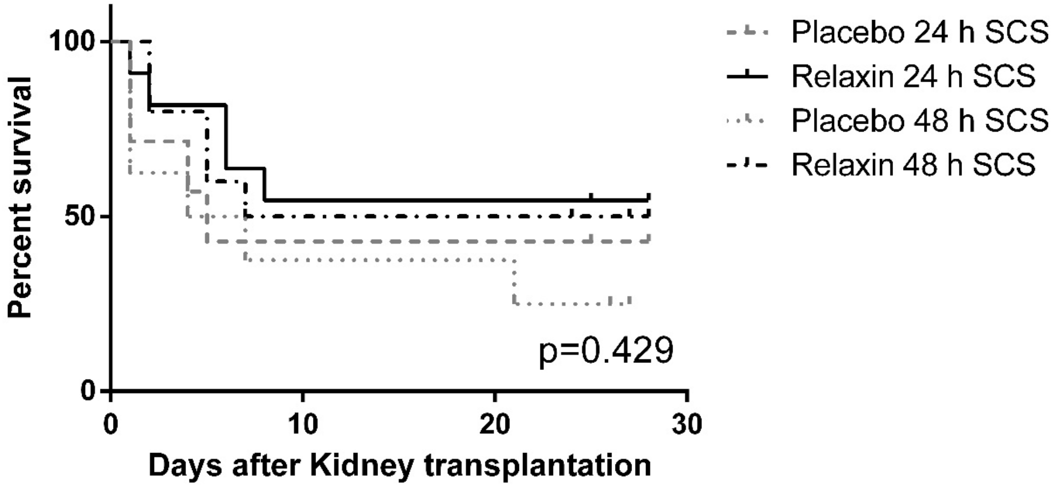

Figure S4. Graft survival after kidney transplantation in Placebo and Relaxin subgroups stored for 24 and 48 hours.

2.2 High vs. low concentration of RLX

Oxidative stress-related genes (Figure S5) and apoptosis, necroptosis-related genes (Figure S6) expression, immunohistochemistry (Table S2), graft function representing parameters (Figure S7) and graft survival (Figure S8) were similar across the animals treated by 5 or 20 nM RLX.

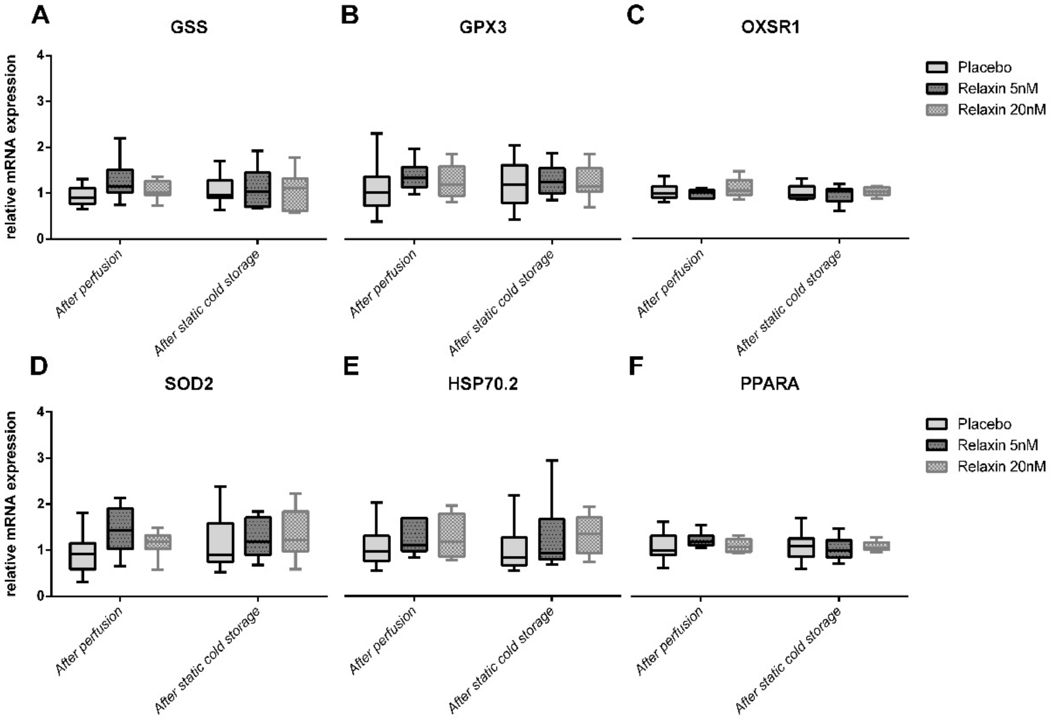

Figure S5. Oxidative stress-related genes expression in kidney tissue after perfusion and static cold storage in Placebo, Relaxin 5nM and Relaxin 20nM subgroups. GSS: Glutathione Synthetase; GPX3: Glutathione Peroxidase 3; OXSR1: Oxidative Stress Responsive Kinase 1; SOD2: Superoxide Dismutase 2; HSP70.2: Heat Shock Protein 70.2; PPARA: Peroxisome Proliferators Activated Receptor Alpha

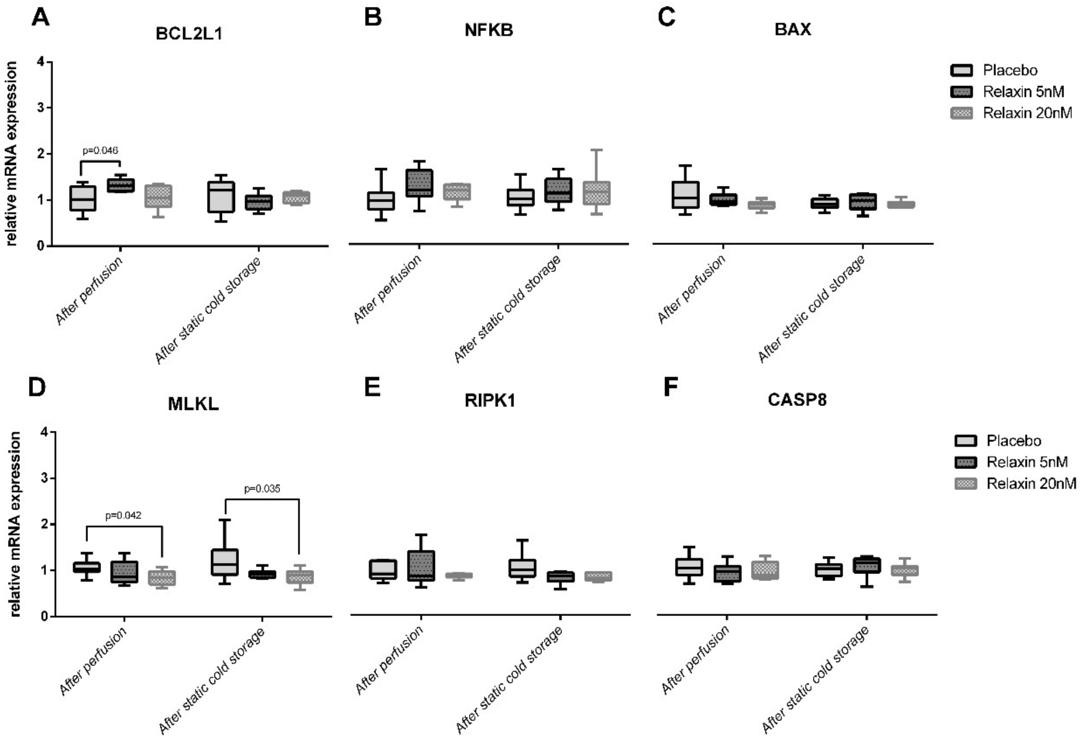

Figure S6. Apoptosis and necroptosis related genes expression in kidney tissue after perfusion and static cold storage in Placebo, Relaxin 5nM and Relaxin 20nM subgroups. BCL2L1: BCL2 Like 1; NFKB: Nuclear Factor of Kappa Light Polypeptide Gene Enhancer In B-cells; BAX: BCL2 Associated X Protein; MLKL: Mixed-lineage kinase domain-like protein; RIPKI1: Receptor Interacting Serine/Threonine Kinase 1: CASP8: Caspase 8.

		Caspase 3				Myeloperoxidase			
		RLX 5nM	RLX 20nM	Placebo	p value	RLX 5nM	RLX 20nM	Placebo	p value
Kidney	After perfusion	0 (0; 0)	0 (0; 0)	0 (0; 0)	*0.999*	0 (0; 0)	0 (0; 0)	0 (0; 0)	*0.999*
	After SCS	0.5 (0; 1)	0 (0; 0)	1 (0; 1.6)	*0.001*	0.1 (0; 1)	0 (0; 0)	1 (0; 1.3)	*0.009*
Renal artery	After perfusion	0 (0; 0)	0 (0; 0)	0 (0; 0)	*0.999*	0 (0; 0)	0 (0; 0)	0 (0; 0)	*0.999*
	After SCS	0 (0; 0)	0 (0; 0)	0 (0; 0.2)	*0.732*	0 (0; 0)	0 (0; 0)	0 (0; 0)	*0.329*
Renal vein	After perfusion	0 (0; 0.3)	0 (0; 0)	0 (0; 0)	*0.449*	0 (0; 0)	0 (0; 0)	0 (0; 0)	*0.999*
	After SCS	0 (0; 0.3)	0 (0; 0)	0.8 (0; 1)	*0.013*	0 (0; 0)	0 (0; 0)	0 (0; 1)	*0.014*
Ureter	After perfusion	0 (0; 0)	0 (0; 0)	0 (0; 0)	*0.999*	0 (0; 0)	0 (0; 0)	0 (0; 0)	*0.999*
	After SCS	0 (0; 0)	0 (0; 0)	0.6 (0; 1)	*0.002*	0 (0; 0)	0 (0; 0)	0 (0; 0)	*0.626*

Table S3. Immunohistochemical evaluation for activated Caspase-3 positivity as well as Myeloperoxidase positivity in kidneys after perfusion and static cold storage in Placebo, Relaxin 5nM and Relaxin 20 nM subgroups. Values are median (Quartile 1; Quartile 3);

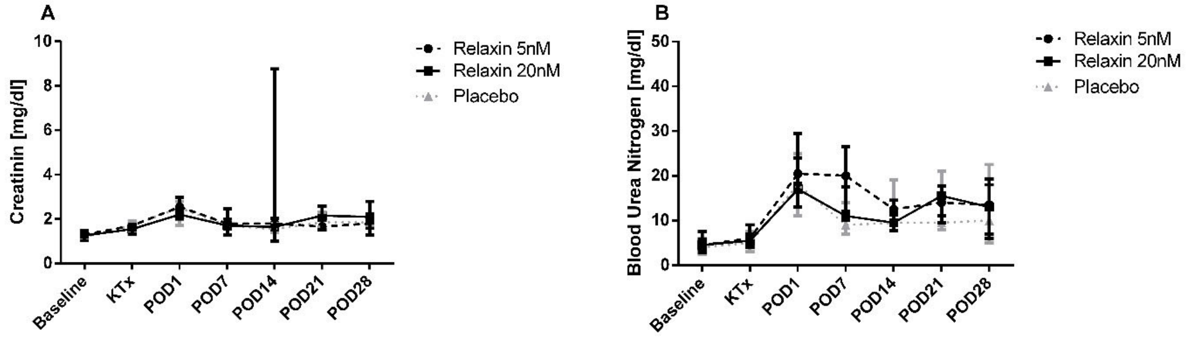

Figure S7. Plasma creatinine (A) and blood urea nitrogen (B) levels after kidney transplantation in Placebo, Relaxin 5nM and Relaxin 20nM subgroups. POD: postoperative day.

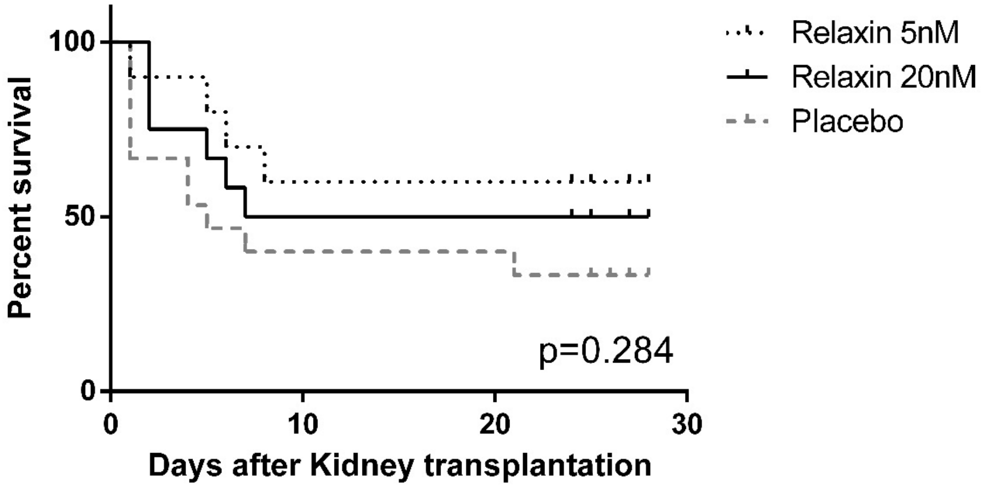

Figure S8. Graft survival after kidney transplantation in Placebo, Relaxin 5nM and Relaxin 20nM subgroups.
